# Induction of virulence factors in *Giardia duodenalis* independent of host attachment

**DOI:** 10.1038/srep20765

**Published:** 2016-02-12

**Authors:** Samantha J. Emery, Mehdi Mirzaei, Daniel Vuong, Dana Pascovici, Joel M. Chick, Ernest Lacey, Paul A. Haynes

**Affiliations:** 1 Department of Chemistry and Biomolecular Sciences, Macquarie University, North Ryde, NSW 2109, Australia; 2 Microbial Screening Technologies, Pty, Ltd, Smithfield, NSW 2165, Australia; 3 Australian Proteome Analysis Facility (APAF), Macquarie University, North Ryde, NSW, 2109, Australia; 4 Department of Cell Biology, Harvard Medical School, Boston, Massachusetts, USA

## Abstract

*Giardia duodenalis* is responsible for the majority of parasitic gastroenteritis in humans worldwide. Host-parasite interaction models *in vitro* provide insights into disease and virulence and help us to understand pathogenesis. Using HT-29 intestinal epithelial cells (IEC) as a model we have demonstrated that initial sensitisation by host secretions reduces proclivity for trophozoite attachment, while inducing virulence factors. Host soluble factors triggered up-regulation of membrane and secreted proteins, including Tenascins, Cathepsin-B precursor, cystatin, and numerous Variant-specific Surface Proteins (VSPs). By comparison, host-cell attached trophozoites up-regulated intracellular pathways for ubiquitination, reactive oxygen species (ROS) detoxification and production of pyridoxal phosphate (PLP). We reason that these results demonstrate early pathogenesis in *Giardia* involves two independent host-parasite interactions. Motile trophozoites respond to soluble secreted signals, which deter attachment and induce expression of virulence factors. Trophozoites attached to host cells, in contrast, respond by up-regulating intracellular pathways involved in clearance of ROS, thus anticipating the host defence response.

Our understanding of how *Giardia* causes disease is incomplete, particularly concerning the early stages of trophozoite pathogenesis^1^. *Giardia* trophozoites attach strongly to the intestinal epithelial cells via a ventral adhesive disc and cause significant damage and disruption to gastroepithelial cells in the absence of cell invasion, secreted toxins and overt inflammation^2^. The interplay between the host and the parasite on establishment is a gap in our knowledge. Recently, host-parasite interaction models with human intestinal epithelial cells (IEC) *in vitro* have provided a foundation for understanding disease induction by *Giardia* trophozoites. Results indicate that these interaction models are stimulatory, inducing expression of parasite factors which have limited or no expression in axenic culture alone^3^. Additional studies have addressed gene expression and transcriptional changes in *Giardia* trophozoites co-incubated with Caco-2 and HCT-8 cells^4^, and HT-29 cells^5^, and analysed the secreted proteomes^3^. There have also been complementary studies of transcripts from IECs exposed to *Giardia* trophozoites^6^,^7^. Together, these studies indicate the efficacy of *in vitro* models to explore the induction of Giardiasis.

Proteomics is one of the few exploratory tools available to understand parasite biology at a physiological level^8^. Currently there are a limited number of proteomic studies performed on *Giardia*^9^,^10^,^11^,^12^,^13^,^14^,^15^, but these do not focus on the host-parasite interplay at key events in pathogenesis. The present study addresses the dynamics of the origin of the infective cycle of attachment during the early stages of pathogenesis. This was achieved using tandem mass tag (TMT) labelling of trophozoite proteins after *in vitro* exposure to host cells during co-incubation (CI) and host secretions (host soluble factors (HSF)) (Fig. 1, Part A). TMT labelling is a quantitative proteomics technique that uses multiplexed isobaric tags which allow greater parallelisation without increasing analysis complexity^16^ (Fig. 1, Part B). This is the first instance of TMT labelling in *Giardia* and demonstrates its sensitivity for protein quantitation for parasite proteomics, even for the subtle changes in protein expression which can occur during short incubation periods.

In this study we have utilised a cell-free incubation, with only soluble products from host target cells, which has facilitated discovery of the very early, attachment independent, stage of *Giardial* pathogenesis. Using HT-29 cells as an *in vitro* model, we have demonstrated that preceding host attachment, trophozoites are actively responding to secreted soluble host signals, and activating manifestly different mechanisms to those involved with attaching to the host. Our data supports the hypothesis that the early stages of Giardial pathogenesis involve a distinctive biphasic process which involves induction of virulence factors in the trophozoites, independent of attachment to the host cells.

## Results

### *In vitro* co-incubation is an active model for trophozoite-host attachment

Comparisons of the rates of adherence of trophozoites to either empty flasks or HT-29 cell monolayers can be viewed in Fig. 2, and the complete dataset can be found in Supplementary Data S1. High rates of adherence occur in the first two hours, with no significant difference in the number of free trophozoites occurring between monolayer co-incubation and control flasks. In the first hour, 78.2% and 72.4% of trophozoites remain free in the media in co-incubation and control flasks, respectively, and this decreases to 56.7% and 61.7% free trophozoites by the second hour (Fig. 2A). After 2 hours adherence plateaus, with only 10% more trophozoites adhering to the flasks in the control between 2–6 hours. However, during co-incubation with HT-29 IEC, attachment increases significantly after 2 hours, with 70% of trophozoites attached after 6 hours. Differences between free and attached trophozoites between co-incubation and control flasks are statistically significant (p < 0.05) beyond 2 hours to assay completion at 6 hours (Fig. 2A). This highlights the viability of *in vitro* host-parasite models, which prompt active attachment that is distinct and separate from adherence.

Further evidence of active interaction during *in vitro* co-incubation is exemplified by changes induced in HT-29 cell morphology over the 6 hour co-incubation. Figure 2B shows that HT-29 cells become enlarged and amorphous by 2 hours, and at later time points detachment from the monolayer has begun to occur (denoted by arrows). Over the 6 hours, increased cellular debris from damaged host cells also accumulated, providing further evidence of pathogenic effects from the interaction with trophozoites.

### Host secretions trigger a non-attaching trophozoite phenotype

The complete results for rates of adherence and host-cell attachment between control and HSF-exposed trophozoites are shown in Fig. 3, with the full dataset in Supplementary Data S1. Trophozoites exposed to HSF showed remarkably reduced rates of both adherence to T 75 cm^2^ flasks (Fig. 3A), as well as host-cell attachment to HT-29 cells during co-incubation (Fig. 3C). The differences in the numbers of adhered/attached trophozoites were significantly lower (p < 0.05) at every time point for both conditions, indicating the switch in phenotype was immediate and sustained. Trophozoites exposed to HSF during conditions for adherence had a consistent average reduction of 45.7% (standard deviation ±1.4%) compared to controls across all hourly timepoints. Trophozoites in control flasks had an average adherence of 49.7% at 6 hours compared to 26.6% in HSF-exposed trophozoites, meaning 46.5% less trophozoites had adhered. During the second round of co-incubation, trophozoites in control flasks of only DMEM had reduced viability, likely due to oxygen tension on parasites in the absence of IECs^4^. This manifested as a 7.6% decrease in adherence between 5 and 6 hours in controls (Fig. 3C). Regardless, differences between adhered controls and host-cell attached trophozoites exposed to HSF also peaked at an average of 20.3% between 3–5 hours in the second round. HSF-exposed and unexposed trophozoites co-incubated with HT-29 showed similar trends of increasing attachment, with similar linear slopes distinct from the trend of exponential plateau observed in the control flasks (Fig. 3C). This suggests HSF-exposed trophozoites are still capable of host-cell attachment, albeit at lower rates. Co-incubations in the presence of HSF reduced host-cell attachment between 9.0% and 24.4% after 1 to 6 hours respectively, with 47.2% less trophozoites attached in co-incubations with HSF after 6 hours. This indicates that HSF produce similar reductions in both adherent and host-attaching populations.

Reduction in the rate of adherence in the first 6 hours was readily observed microscopically (Fig. 3B). The trophozoites that adhered in the presence of HSF were also semi-motile on the flask wall compared to trophozoites in controls, which maintained their position after settling and adhering (Supplementary Media 1). This indicates a reduced proclivity for adherence, further supported by observations during detachment from flasks, with controls containing only DMEM requiring twice the chilling time on ice as well as additional vortexing to liberate trophozoites before the second round of the assay. Trophozoites exposed to HSF showed no reduction in viability and remained motile throughout the entire assay.

### Quantitative proteomics

The complete TMT dataset, including protein identifications, label ratios and peptide information can be viewed in Supplementary Data S2. A non-redundant total of 1664 proteins from *G. duodenalis* isolate BRIS/95/HEPU/2041 were identified from a non-redundant total of 13 465 peptides (Fig. 4A). Peptide to spectrum matching was performed using the Assemblage A1 genome sequence of isolate WB C6 (ATCC 50803). Previous quantitative proteomic analyses have demonstrated no significant difference in peptide numbers identified in subassemblage A1 isolates when using the WB C6 genome sequence as a database^17^. Similarly, recent comparative genomics analysis demonstrated that the A1 subassemblage is more conserved than the A2 subassemblage, with approximately 7.5 single nucleotide polymorphisms (SNPs) per 100 000 between genome sequences of two A1 isolates^18^, compared to 350 SNPs per 100 000 between two newly sequenced A2 isolates^19^. Both genomic and proteomic data therefore indicates the A1 subassemblage is sufficiently conserved for the WB C6 genome sequence to provide a reference database for quantitative proteomics experiments within this taxonomic group.

**Figure 1:**
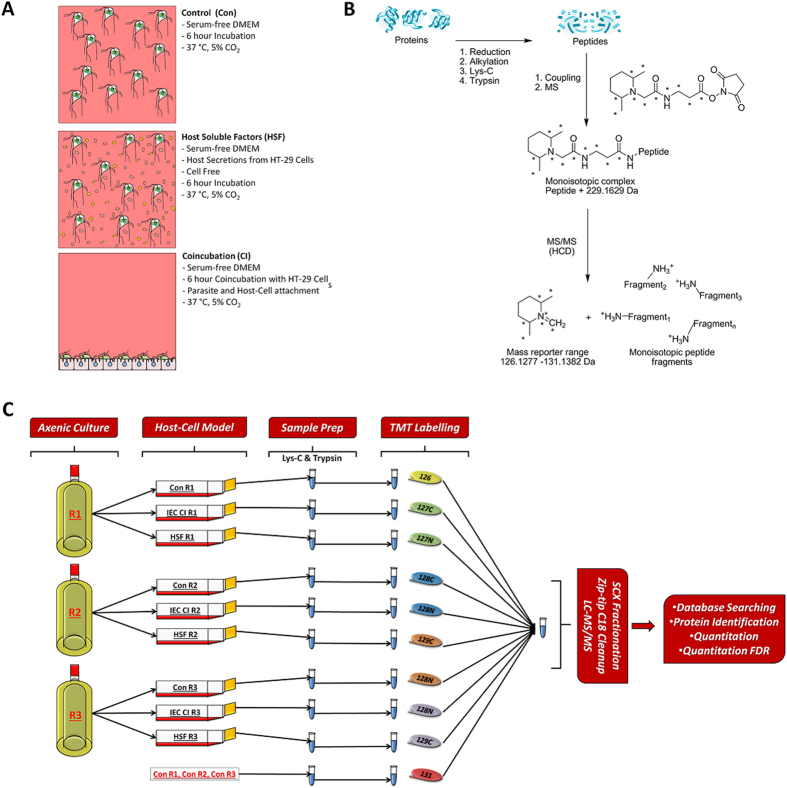
Experimental design and TMT labelling workflow for the experiment. (**A**) Summary of the experiment conditions of the control, host soluble factor and co-incubation treatments. (**B**) Explanation of the TMT-labelling strategy utilised in the experiments. Peptides from the triplicates of the 3 conditions and a pooled control were labeled with one each of the TMT 10plex reagents. These reagents are observed as a monoisotopic complex in the first round of MS analysis on a high resolution mass spectrometer. During MS/MS and HCD based fragmentation, the TMT labels are fragmented to produce 10 reporter ions with distinguishable masses in the low m/z range, which allow relative protein quantitation (**C**) Overarching experimental design and workflow. Biological triplicates of BRIS/95/HEPU/2041 were grown to confluence in parasite culture in TYI-S-33 medium, before replicates were split into a replicate of each control for cell culture conditions (Con), co-incubation with IEC monolayers (CI IEC) and incubation in host soluble factors generated by IECs (HSF). Proteins were extracted from the 9 replicates, and a pooled control was generated from equal aliquots of protein from the control triplicates. After proteolytic digestion, samples were labelled in a 10 plex TMT reaction and then pooled. The combined sample was fractionated by SCX chromatography and desalted using a C18 ZipTip prior to LC-MS/MS on a Q-Exactive Orbitrap.

Quantitation ratios were calculated as biological triplicate values from host-parasite interaction replicates relative to their respective control replicates (Fig. 1C). Proteins were considered differentially expressed above a fold change of 1.2 and below a fold change of 0.8 in addition to a significant p-value of ≤ 0.05^20^ (Fig. 4B). Using a two-stage criterion for differentially expressed proteins greatly improved the statistical confidence, as single-paired t-tests eliminated proteins with high ratio variability between replicates. Statistical evaluation of the dataset by comparing individual control replicates against the pooled control indicated that levels of variability in the entire dataset were very low (Supplementary Methods, Supplementary Figure S1). Principal Component Analysis (PCA) also indicated that the control replicates were both clustered together, and were clearly discriminated from HSF and CI treatments (Supplementary Methods, Supplementary Figure S2, Supplementary Data S3). In addition, the distribution of the p-values indicates an underlying signal of differential protein expression when comparing the HSF and CI treatments to the control replicates (Supplementary Methods, Supplementary Figure S3).

A total of 68 differentially expressed proteins were identified in *Giardia* trophozoites during host-parasite interactions (Fig. 4A), with 45 proteins differentially expressed in trophozoites co-incubated with IECs (Table 1), and 38 proteins in HSF-exposed trophozoites (Table 2). These included several up-regulated proteins previously identified the transcript level during co-incubation of *Giardia* trophozoites with IECs^4^,^5^, including tenascin proteins, cathepsin B precursor, uridine phosphorylase 1 (UPL-1) and thioredoxin. A total of 30 variant-specific surface proteins (VSPs) were also identified in the whole dataset, with eight and four variants up-regulated in HSF and CI treatments, respectively (Supplementary Table 1). This up-regulation of a large number of VSP variants in HSF-exposed trophozoites has not been previously reported for *in vitro* host-parasite models in *Giardia*. The *G. duodenalis* VSP gene family contains both conserved, homologous regions of gene sequence as well as unique regions^21^. Of the eight differentially expressed VSP variants in HSF-exposed trophozoites, five of the protein-level identifications are from non-homologous peptides. Though the remaining three VSP variants have homologous peptides associated with the identification, multiple peptides have been detected for these identifications, and protein-level identification was assigned based on the most likely candidate given the total and composition of peptides matched.

**Figure 2:**
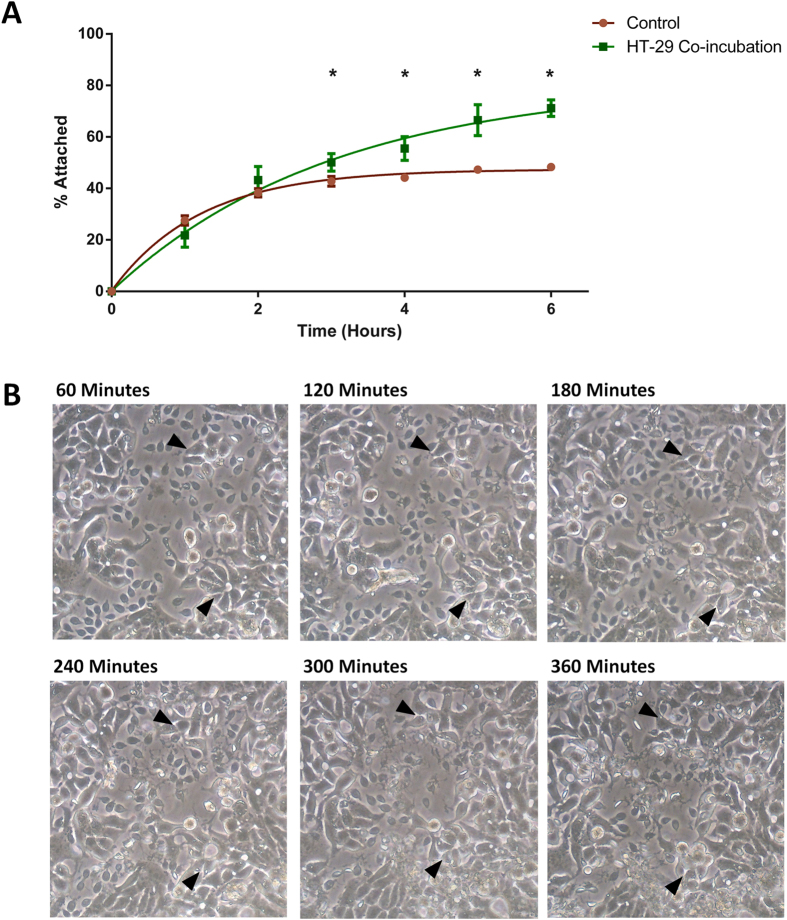
Results of the *in vitro* host-cell attachment versus non-specific adherence. (**A**) Rates of attachment between *Giardia* trophozoites incubated with HT-29 cells over 6 hours against a control for adherence (T75 flasks with media only). A ‘*’ indicates a significant difference in % attached trophozoites compared to control (designated by p-value ≤ 0.05). (**B**) Changes in HT-29 cell morphology induced during co-incubation with *Giardia* trophozoites. The arrows (▲) highlight 2 regions of affected cells throughout the 6 hour co-incubation.

Overall, a non-redundant total of 47 proteins were up-regulated, while 21 were down-regulated during *in vitro* host-parasite interactions. This smaller down-regulated protein response suggests the response of *Giardia* trophozoites to host signals *in vitro* may be inductive rather than repressive. The dataset size of differentially expressed proteins is similar to those seen previously with HT-29 cells over 6 hours incubation in RNA studies^5^. Although some common differentially expressed proteins were observed, the majority of proteins were expressed uniquely between trophozoites stimulated by host secretions compared to attachment (Fig. 4C). The process of washing IEC-monolayers prior to co-incubation, as well as introducing *Giardia* trophozoites to fresh, serum-free interaction media, ensured secreted factors from host IECs were removed at the beginning of co-incubation, and did not establish to sufficient levels during the 6 hour time course. Therefore, there was not the same induction of responses observed in trophozoites incubated solely in the presence of pre-established levels of HSF, and only minimal overlap between the treatments. Expression intensity also varied, with HSF driving a higher fold change than those in trophozoites interacting with the IEC monolayer (Fig. 4B). Lower fold changes in co-incubation are likely due to the few hours delay in host-attachment by trophozoites, as evidenced during the co-incubation attachment assay (Fig. 2A). These results indicate during *in vitro* interactions *Giardia* trophozoites respond at the very early stages to host secretions prior to attachment. These soluble signals from the host stimulate distinct and independent pathways for establishing disease.

### Host secretions induce virulence factors in a motile population

Trophozoites incubated in HSF up-regulated production of 25 proteins (Table 2), the majority of which were membrane-associated or secreted. A total of 11 up-regulated proteins contained evidence of secretion or transmembrane helices in one or more predictive algorithms (Supplementary Figure S4). These 11 proteins consisted of VSP variants, tenascins, cathepsin B and high cysteine membrane protein (HCMP) (Supplementary Data S4). A total of 8 VSPs were up-regulated in the 6 hours, which constitute 32% of all up-regulated proteins in trophozoites incubated in HSF, and 26.7% of all VSP variants identified. While multiple HCMP variants have been previously reported during *in vitro* host-parasite interactions at the transcript level^4^,^5^, the up-regulation of multiple VSPs has not been previously observed in RNA studies. There were 4 VSP variants identified in trophozoites co-incubated with IECs, but lower fold changes in these VSPs suggest that this is likely due to re-establishment of host-soluble signals throughout the experimental timecourse. Five of the 8 VSPs were predicted as secretory as well as possessing transmembrane helices, while 2 lacked prediction for secretion but contained transmembrane helices, and one met no predicted secreted or membrane criteria (GL50803_98861). Expression of both tenascin precursor (GL50803_8687) and tenascin X (GL50803_14573) was increased in trophozoites exposed to HSF, which is consistent with previous observations of increased expression in tenascin gene transcripts during *in vitro* host-parasite interactions^5^. Tenascin proteins share similar domains to the membrane-associated VSPs, but lack transmembrane helices (Supplementary Data S4 and S5). Tenascin X also possesses an additional EGF-like extracellular domain (IPR013111). In light of functional and predictive bioinformatics, it is highly likely that these proteins are secreted. Exposure of trophozoites to HSF also resulted in up-regulation of the cysteine protease cathepsin B (GL50803_16779), which is consistent with previous RNA studies^4^,^5^. The *G. duodenalis* cystatin homologue^22^ was up-regulated in trophozoites exposed to HSF and during co-incubation, indicating sensitivity to multiple host signals. This cystatin (GL50803_27918) is currently unannotated, with a single ortholog in all *G. duodenalis* genomes, and lacks GO and Interpro annotations whilst displaying low sequence homology to other parasite and eukaryotic cystatins. However, sequence analysis indicates conserved crucial residues, including a glycine in the N-terminal region, and hydrophobic residues in both first and second binding loops which forms the wedge that inserts and deactivates the active site of cathepsin proteases^23^. A putative thioredoxin (GL50803_3910) was also up-regulated, consistent with potential oxygen stress in cell culture conditions, and data from previous studies^4^,^5^. The single *Giardia* 14-3-3 homologue (GL50803_6430) was also up-regulated, which is involved in binding to signal proteins involved in phosphorylation cascades^24^. There were five up-regulated proteins which were only annotated as ‘hypothetical proteins’ (GL50803_16693, GL50803_14278, GL50803_17340, GL50803_3755, GL50803_2012), and for which there are no current GO annotations or Interpro protein structure and fold information. Interestingly, none of these proteins have been previously reported as differentially expressed in transcriptomic *G. duodenalis in vitro* host-parasite interaction models.

### Host-cell attachment prompts intracellular anticipation of host defences

A total of 34 proteins were up-regulated in trophozoites co-incubated with the IEC monolayer (Table 1). In contrast to trophozoites incubated with soluble host signals, there were only four secreted and membrane proteins, all of which were VSPs, indicating no new secreted or membrane protein classes were detected (Supplementary Data S5). A single protein (GL50803_16188), with annotations associated with chromosome organisation, was localised to the nucleus, meaning 29 (85%) of the up-regulated proteins are likely to be localised to the cytosol (Supplementary Figure S5). Of these up-regulated proteins, five were annotated with oxidoreductase functions (GL50803_5810, GL50803_3042, GL50803_114609, GL50803_10358, GL50803_4946), which include functions for oxidative defence and reactive oxygen species (ROS) detoxification, as well as production of pyridoxal phosphate (PLP). These five proteins constitute 9.8% of all annotated oxidoreductases in the *G. duodenalis* A1 genome. GL50803_5810 is currently unannotated but is most likely to be the *Giardia* homolog for pyridoxamine-phosphate oxidase, the enzyme responsible for the rate-limiting reaction in the production of the active form of coenzyme vitamin B_6_, due to the singular presence of these functional protein domains in the genome. Concordantly, GL50803_480 was also upregulated, which has gene ontology (GO) and Interpro annotations associated with deaminase activity in converting reactive enamine/imine intermediates in PLP-dependent enzyme reactions. Furthermore, GL50803_29708 is a hypothetical protein with annotations for pyridoxal-phosphate dependent aminotransferase. Additionally, several proteins in the ubiquination pathway were also up-regulated, including ubiquitin (GL50803_7110), an ubiquitin carrier enzyme (UBCE) (GL50803_3171) and ubiquitin-conjugating enzyme E2-17 (GL50803_15252). The cystatin homologue (Gl50802_27918) was up-regulated, as also observed during exposure to host soluble factors. UPL- 1was also up-regulated, which has been previously been reported in trophozoites co-incubated with HT-29 cells^5^. Again, the *Giardia* 14-3-3 homologue (GL50803_6430) was up-regulated as seen in HSF-exposed trophozoites, along with another similar tetratricopeptide (TPR) containing protein (GL50803_10529). There were seven hypothetical proteins that lacked GO and Interpro functional and structural information (GL50803_16693, GL50803_113415, GL50803_3345, GL50803_2267, GL50803_14567, GL50803_9506, GL50803_15039), none of which have been previously reported in earlier transcriptomic studies of *in vitro* host-parasite interaction models for *G. duodenalis.*

## Discussion

**Figure 3:**
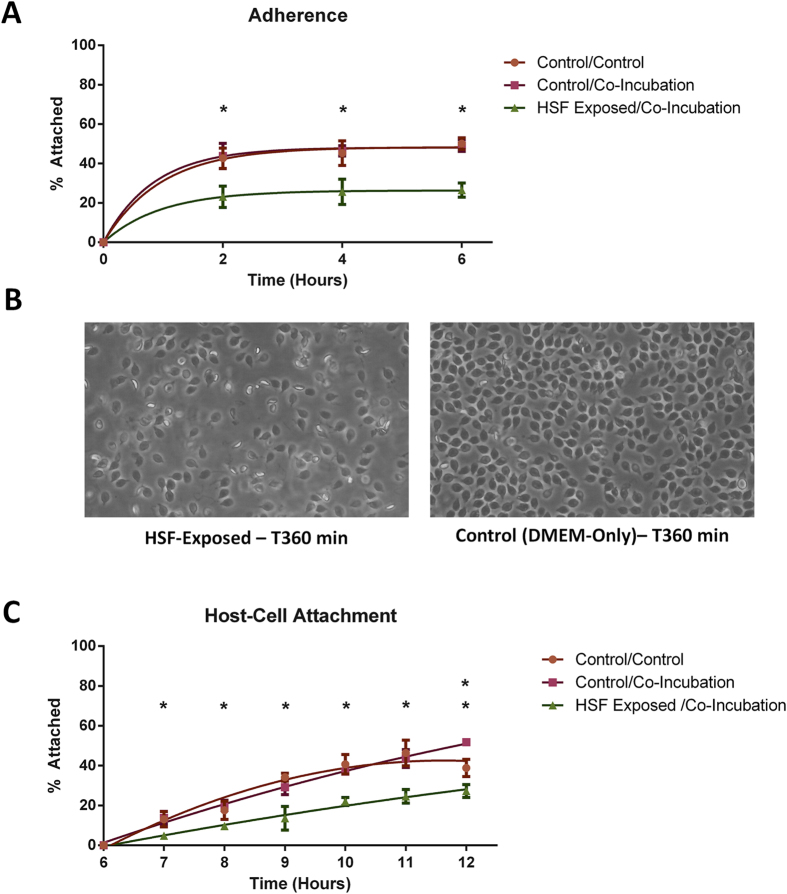
Results for the effects of HSF on adherence and host-cell attachment during co-incubation. The 3 treatments are as follows: trophozoites in serum-free DMEM in the first round followed by co-incubation with HT-29 (Con/CI), trophozoites exposed to HSF in serum-free DMEM and then co-incubated with HT-29 in the presence of HSF in the second round (HSF/CI) and a control of serum free DMEM in both rounds of the assay (Con/Con). Trophozoites were transferred from the first round of adherence to the second round of co-incubation. (**A**) Rates for adherence during the first 6 hours between trophozoites co-incubated in T75 flasks containing serum-free DMEM (Con/CI, Con/Con) and trophozoites incubated in the presence of HSF (HSF/CI). A ‘*’ indicates a significant difference in % attached trophozoites (designated by p-value ≤ 0.05). Rates of adherence in HSF-exposed trophozoites were statistically significantly lower at all 3 timepoints compared to unexposed trophozoites. (**B**) Images depicting the differences in density of adhered trophozoites in HSF-exposed and HSF-free flasks after 6 hours of incubation. Flasks shown were seeded with the same number of trophozoites. (**C**) Rates of host-cell attachment in the second 6 hours of the assay. Rates of host-cell attachment between trophozoites exposed to HT-29 monolayers (Con/CI) are compared to trophozoites incubated with HT-29 monolayers in the presence of HSF (HSF/CI). A control for adherence was also performed in triplicate in serum DMEM without HT-29 cells. A ‘*’ indicates a significant difference in percentage of attached trophozoites compared to control (designated by p-value ≤ 0.05). Rates of attachment in HSF-exposed cells was significantly lower at all time points between both the control for adherence, and the rate for host-cell attachment in unexposed trophozoites. The number of attached trophozoites co-incubated with HT-29 monolayers without HSF was statistically significantly higher compared to the control for trophozoite adherence in flasks only after 6 hours incubation.

*In vitro* host-parasite models in *G. duodenalis* are designed to replicate trophozoite attachment to host cells and thereby induce expression of virulence and disease factors. Previous studies have demonstrated co-incubation induces secreted proteins^3^ and expression of gene transcripts demonstrably different from constitutive expression in culture^4^,^5^. We have shown co-incubation with cell monolayers permits attachment of trophozoites to host cells, which is significantly different from trophozoite adherence to flasks alone (Fig. 2). Further, our results demonstrate that *Giardia* induces proteins upon exposure to either host cell products, or upon attachment to host cells, and the proteins expressed in these responses are independent and distinct (Fig. 5). We have also demonstrated a unique sensitivity in trophozoites to secreted products from host cells, and that HSF-exposed trophozoites switch to a non-attaching motile phenotype (Fig. 3). Significantly, exposure to these host secretions was sufficient for induction of virulence factors in trophozoites. To our knowledge, this is the first demonstration of an interactive, biphasic process during early pathogenesis which shows a clear difference between motile trophozoites responding to host soluble signals, and trophozoites attached to host-cells.

We have provided experimental evidence that exposure to HSF results in up-regulation of membrane and secreted proteins prior to attachment in *Giardia* trophozoites (Fig. 5). Several of these induced proteins are also virulence factors, and we have demonstrated their sensitivity to host secretions in the absence of host cells. VSPs are a gene family and collective virulence factor responsible for immune evasion and antigen variation^21^. A single VSP is expressed on the trophozoite surface at any given time, though multiple variants accumulate in culture in the absence of immune selection. On exposure to HSF a quarter of all expressed VSP variants were up-regulated, which constituted over a third of observed up-regulated proteins. These changes in VSP expression likely represent selection of favourable variants for host pathogenesis or virulence. Interestingly, four up-regulated VSPs possessed additional functional domains to the core VSP protein domains (Supplementary Data S4). These included the metallopeptidase domain homologous to the virulence factor leishmanolysin from the parasite *Leishmania*^25^,^26^, and the BmKX domain found in scorpion toxins^27^. This suggests individual VSP variants may act as independent virulence factors beyond their role collectively in immune evasion^28^. The magnitude of differential expression in VSPs has not been reported previously in *Giardia* host-parasite interactions. This may be due to the absence of host secretions in interaction media, or possibly the virulent phenotype of BRIS/95/HEPU/2041^29^,^30^, which features diverse VSP variant repertoires in culture^12^,^17^. In addition to previous studies showing VSP switching elicited by specific monoclonal antibodies^31^,^32^, we present here experimental evidence suggesting soluble host factors may drive antigen switching events.

Cathepsin B is a confirmed virulence factor and secreted protein which degrades IL-8 and inhibits neutrophil chemotaxis^33^,^34^. Exposure to secreted host factors up-regulated cathepsin B as well as cystatin, a protease inhibitor of cathepsins. When secreted, cystatins potentially modulate host immune response, as observed in parasitic nematodes^35^,^36^, but may also internally regulate parasite cathepsins^37^. The *Giardia* cystatin is phylogenetically basal to eukaryotic cystatins and more closely related to bacterial equivalents^22^,^26^, which makes it difficult to extrapolate its internal or external targets^36^.

Tenascins share similar ‘EGF-like’ (IPR000742) domains to VSPs, and are glycoproteins involved in cell-to-cell adhesion in mammals and chordates, with unknown functions in parasites and early eukaryotes^38^,^39^. Interestingly, research in mammals demonstrates that tenascins bind and interact with lectin domains^40^,^41^, which may have implications for parasite interactions with molecules involved in mammalian innate immunity. Significantly, both transcript and protein data reproducibly identify tenascins in host-*Giardia* interactions, and our bioinformatics analysis demonstrated for the first time that these are possibly secreted proteins during early pathogenesis.

**Figure 4:**
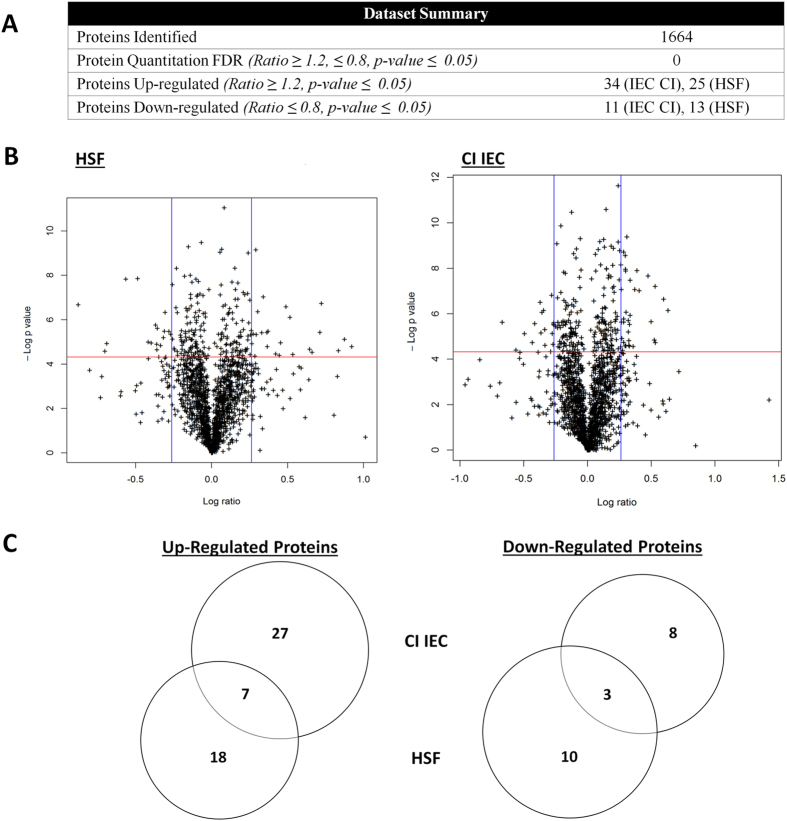
Protein identification and protein quantitation summary from TMT labelling of trophozoites co-incubated with the IEC monolayer (CI IEC) and with host soluble factors alone (HSF). (**A**) Outline of protein identification, differentially expressed proteins and protein quantitation FDR for the dataset. (**B**) Volcano plots illustrating the dual criteria for differentially expressed proteins. The x-axis represents log fold change with the vertical blue lines indicating 1.2 and 0.8 ratio, while the -log p value is plotted on the y-axis with proteins above the red horizontal line indicating significance ≤0.05. Each data point represents a single identified protein. Proteins within the upper and outer quadrants meet both the fold change and p-value cut-off, and are therefore considered as differentially expressed. (**C**) Proportional venn diagrams showing overlap between up-regulated and down-regulated proteins in trophozoites between CI IEC and HSF treatments.

**Figure 5:**
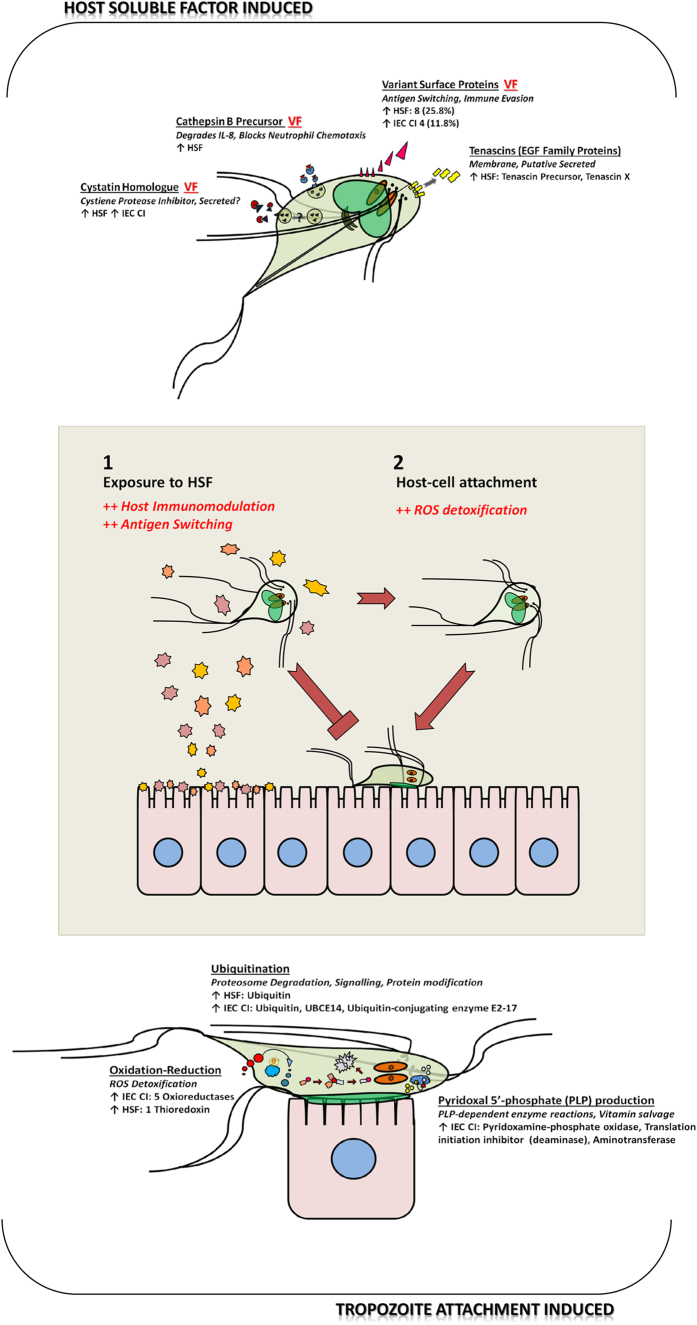
Figure depicting the biphasic model of interaction between *G. duodenalis* trophozoites and host-cells proposed in our paper. Proteins trends, families and pathways induced during host-parasite interactions in *Giardia* are distinguished between HSF-induced in non-attached, motile trophozoites (above) separate to cascades induced in host-cell attached trophozoites (below). ‘VF’ indicates induced protein groups related to known or putative virulence factors in *Giardia*, which were induced by host secretions. The middle of the figure shows the two distinct stages observed in early pathogenesis, where host-soluble factors lead to a switch to a non-attaching, motile population phenotype. Motile trophozoites migrate further through the gastrointestinal tract, where in the absence of host soluble factors and more optimum conditions, *Giardia* attaches to host cells. These two stages of host-parasite interactions induce distinct and independently protein responses.

Trophozoites exposed to host secretions displayed a non-attaching phenotype (Fig. 3) and a distinct protein response from co-incubated trophozoites (Fig. 4). In *Giardia*, attachment occurs via its ventral disk through cytoskeletal mechanisms related to microtubules, but also via *Giardial* lectins^42^. Earlier attachment assays demonstrate several factors that decrease attachment, including temperature, acidification, osmolality and tonicity, incubation with lectins, exposure to lectin-binding carbohydrates and, most prominently, interference with contractile filaments^42^,^43^,^44^. None of these factors account for the reduction in attachment observed in HSF-exposed trophozoites during our experiments. Previous investigation of host factors is limited to trypsin, which produced minor reductions in attachment^42^, or exogenous or host lectins, which are associated with trophozoite agglutination^44^, which did not occur during our assay. Our results indicate a unique factor may be responsible for non-attachment in trophozoites exposed to host secretions. In addition, our results also support the hypothesis that host secretions promote an active switch to a motile population, potentially to continue migration through the gut to a less ‘hostile’ environment, as has been previously observed in gastrointestinal nematodes which relocate in response to localised host immune responses^45^. If one assumes that host secretions contain immune molecules involved in parasite clearance, such as cytokines^46^, up-regulating VSPs, for immune evasion, and cathepsin B and cystatins, for immunomodulation^28^, are consistent with the hypothesis that host secretions induce proteins to counteract host immunity in a motile *Giardia* population seeking ideal conditions for attachment (Fig. 5).

In contrast, trophozoites co-incubated with IECs induced intracellular pathways in anticipation of non-specific immune responses to infection. The lower intensity of observed protein expression fold changes coincides with the delay observed between adherence to control flasks and adherence to host cell monolayers (Fig. 2A). Co-incubation induced up-regulation of proteins for ubiquitination, including two E2 carrier/conjugating enzymes as well as the ubiquitin moiety. Though ubiquitination pathway and enzymes are simpler in *Giardia* compared to mammals^47^, it has been shown to play an important role during differentiation, with modifications on a diverse range of proteins^48^. In our results, an increase in ubiquitin-modified proteins is likely to indicate increased proteasome activity in response to rapid transition from axenic culture to host-parasite interaction.

Parasite co-incubation with host cells prompted up-regulation of a diverse range of oxidoreductases highlighting the importance of ROS detoxification in parasite establishment and survival. Trophozoites exposed to host-soluble signals up-regulated thioredoxin for oxidative defense in increased oxygen during cell-culture conditions, but not oxidoreductases. Co-incubation with host IECs in aerobic cell culture conditions provides some protection against oxidative stress in *Giardia*, but defence genes are still up-regulated at the transcriptomic level^4^,^5^. As trophozoites incubated in cell-free interactions have greater environmental oxygen stress, this up-regulation of oxidoreductases is likely to be a specific response to interaction with host cells which are known to produce exogenous ROS in response to gastroepithelial parasites^49^. Similarly, production of reactive nitrogen species (RNS) negatively impacts *Giardia* growth, differentiation and viability. Therefore, trophozoites inhibit RNS production by outcompeting host cells for substrates by rapid arginine consumption^50^, highlighting the importance of this pathway in pathogenicity and virulence^7^,^51^. Earlier transcriptomic studies have confirmed IECs co-incubated with *Giardia* up-regulate genes associated with ROS and RNS production^6^,^7^, and our results reinforce the necessity for *Giardia* to counteract these host defences. A flavo-diiron protein (GL50803_10358) with very low nitric oxide (NO) reductase activity but remarkably high O_2_ detoxification activity, which has been shown experimentally to promote parasite survival in the small intestine^52^, was up-regulated in our experiments. This was accompanied by a cumulative up-regulation of three other oxidoreductases with functions in removal of ROS, indicating that co-incubation either prompts, or trophozoite attachment anticipates, ROS production in host cells^6^.

Pyruvate flavodoxin oxidoreductase (PFOR) converts pyruvate to acetyl coenzyme A during anaerobic energy production^53^ but is also up-regulated during oxidative stress and involved in the antioxidant system^49^,^54^. Similarly, peptide methionine sulphoxide reductase (Msr) (GL50803_4946) is likely to alleviate oxidative stress by reversing oxidation of critical methionine residues which might otherwise cause protein inactivation^55^. Following this trend, an iron-sulphur (FeS) containing hybrid cluster protein (GL50803_3042) was also up-regulated, which is a member of a protein class implicated in defence against oxidative stress in bacteria, Archaea and protozoans including *Trichomonas vaginalis*, and *Entamoeba histolytica*^56^. Collectively, these reinforce the necessity of maintaining redox homeostasis in the face of ROS produced by host defences, with multiple oxidoreductases induced by trophozoites soon after first contact with IECs. The remaining up regulated oxidoreductase is the *Giardial* pyridoxamine-phosphate oxidase homolog, which is the only protein annotated with this functional domain (GO:0004733) with a single orthologue in all sequenced *G. duodenalis* genomes.

Two PLP-associated enzymes were also up-regulated, suggesting PLP-regulated enzymes and pathways play a role in disease induction. The production of PLP, its biological role in *Giardia*, and its antioxidant properties are all unknown, although PLP-dependent enzymes have been suggested as drug candidates for multiple protozoans^57^. Importantly, crucial PLP-dependent cysteine desulfurases in *Giardia* possess conserved PLP-binding residues and are involved in FeS cluster biosysnthesis^58^. Additionally, PLP-dependent enzymes are involved in polyamine biosynthesis in *Giardia*, and it has been demonstrated that specific inhibition of ornithine decarboxylase results in interruption of this biosynthesis and eventuates in parasite death^59^. PLP-dependent pathways and enzymes have been wholly unexplored in *Giardia* for therapeutics, and provide a potentially novel pathway for blocking disease induction.

In *Giardia*, analysis of pathogenesis has emphasised the importance of trophozoite attachment to host-cells. However, we have demonstrated for the first time that trophozoites respond independently to host soluble signals early in pathogenesis, and initial exposure to these secretions prompts a switch to a motile population phenotype. We hypothesise protein expression induced by host secretions is aimed at counteracting host immune defences while a motile population migrates to an optimum environment for attachment. In this biphasic model, trophozoites are either initially sensitised to host soluble signals, or undergo host attachment and induce proteins in advance of host defences (Fig. 5). Anticipating the production of molecules from the host cells which are designed to clear parasite infection, trophozoites attached to host cells produce a wide range of oxidoreductases for neutralising exogenous ROS. These trophozoites also up-regulate proteins associated with PLP production, while increasing ubiquitin/proteasome mediated protein turnover for production of disease related proteins. This dual combination of distinct responses to either host soluble factors, or host attachment, indicates early pathogenesis involves multiple and distinct levels of crosstalk between host and parasite. These are independently regulated and do not require attachment to host-cells for induction of virulence factors. Thus, these host secreted signals are sufficient to induce virulence factor expression in *Giardia* cells, which occurs independent of parasite attachment to host cells.

## Methods

### Cell and *Giardia* cultures

Human intestinal epithelial cell line HT-29 were grown in high glucose DMEM containing GlutaMAX™ (Gibco, Life Technologies) supplemented with 10% Foetal Bovine Serum (FBS) (Gibco, Life Technologies) and 1% Penicillin/Streptomycin (5000 U/mL) (Gibco, Life Techologies). HT-29 cells were maintained in 75 cm^2^ flasks (Corning Incorporated, New York) and sub-cultured 3 times a week in an incubator at 5% CO_2_ at 37 °C. *Giardia* trophozoites of BRIS/95/HEPU/2041 were grown in TYI-S-33 medium supplemented with 10% newborn calf serum and 1% unfractionated bovine bile^60^. Parasites were subcultured at end-log phase into fresh media and interaction studies were carried within 5 passages from recovery from cryopreservation. Absence of bacterial and fungal contamination was verified using serial dilutions and nutrient agar Petri plates to ensure no colony forming units were detected in either human or parasite cultures prior to interaction and protein extraction.

### *In vitro* interaction and co-incubation

HT-29 cells were grown in 75 cm^2^ flasks to confluence prior to interaction studies, and washed twice with 37 °C PBS to remove media and serum traces. In order to generate the HSF fraction for interaction, confluent monolayers of HT-29 were incubated for 20 hours in serum-free, DMEM media. Cell viability and monolayer integrity was monitored throughout the 20 hour incubation and final cell viability measured through trypan blue dye exclusion (Sigma Aldrich). Co-incubated media was decanted from the monolayer, centrifuged to remove any whole cells or cellular debris and filtered through a 0.22 μm pore filter (Merck Millipore). To normalise the HSF fraction for flask variation, all DMEM generated from confluent monolayers was pooled.

Figure 1 shows the experimental design and workflow of the TMT experiment. *Giardia* trophozoites were grown to mid-log phase in triplicate in ‘inside-out’ custom roller bottles^61^. Trophozoites were washed twice with 37 °C PBS to remove media and serum traces. Trophozoites were both motile and viable at the beginning of host-parasite interactions, and twice the volume of serum-free DMEM used in normal cell culture was used to reduce oxygen tension in all treatments as previously described^4^. HT-29 monolayers, grown in 175 cm^2^ flasks to 100% confluence, were washed with PBS prior to interaction to remove HSF to minimise overlap between interaction studies. For co-incubation with the HT-29 monolayer, cells were incubated in a 3:1 ratio in serum-free DMEM. For incubation with HSF, trophozoites were incubated in the filtered media from confluent HT-29 cells and for the control *Giardia* trophozoites were incubated in serum-free DMEM. Incubation and interactions were performed in triplicate for 6 hours at 5% CO_2_ at 37 °C. Cell and parasite viability was monitored throughout the 6 hours as well as the viability of the HT-29 monolayer and final cell viability quantified through exclusion dye assay using erythrosin B (Sigma Aldrich) and trypan blue for trophozoite and HT-29 cells, respectively, with viability >95% considered acceptable. Trophozoite adherence was also monitored in co-incubation replicates, as was trophozoite motility monitored throughout all treatments. The integrity of the HT-20 monolayer was also observed throughout the co-incubation period.

### *In vitro* attachment assays

To measure rates of *Giardia*-host cell attachment, HT-29 cells were grown in 75 cm^2^ flasks to confluence, and *Giardia* trophozoites were grown to mid-log phase. Co-incubation of trophozoites and HT-29 cells were performed in triplicate as in *in vitro* interaction studies, with serum-free DMEM at twice normal volume with a 3:1 trophozoite to cell ratio. A control for adherence consisted of trophozoites incubated in triplicate in 75 cm^2^ flasks, in serum free DMEM. The assay was performed over 6 hours at 5% CO_2_ at 37 °C, with attachment monitored at hourly time points from T_0_ to T_360_ minutes. Media was sub-sampled and total number of free, unattached trophozoites in the flask counted by haemocytometer in both co-incubation and control triplicate flasks. The total number of free trophozoites was expressed as a percentage of trophozoites at T_0_. Difference in rates of attachment was assessed for statistical significance using an unpaired t-test at each hourly time point, with a p-value ≤ 0.05 considered significant.

A second assay was performed to measure the impact of host soluble factors on both trophozoite adherence to flasks, as well as trophozoite host-cell attachment. The assay conditions were run in triplicate with the same volumes, ratios and media as previously specified. HSF fractions were generated as before. The assay was run over 12 hours, with the first incubation in flasks without cells from T_0_ to T_360_, followed by a second round of co-incubation with HT-29 cells from T_360_ to T_720_. The experimental design of the attachment assay using HSF-exposed trophozoites is shown in Supplementary Figure S5. A total of 3 treatments were analysed; trophozoites in serum-free DMEM in the first round and then co-incubated with HT-29 cells in the second round (Con/CI), trophozoites exposed to HSF in serum-free DMEM in the first round and then co-incubated with HT-29 in the presence of HSF in the second round (HSF/CI). Lastly, a control was run in serum free DMEM without HT-29 monolayers in both rounds of the assay (Con/Con). The same trophozoites were used in both rounds of the assay. Trophozoites were detached from the flask after the first round incubation, and transferred with the media into flasks containing HT-29 for subsequent host-cell co-incubation. The control triplicates from the first round were detached and transferred into fresh flasks for the second round of the assay. Trophozoites were counted every 2 hours in the first round, and then hourly during co-incubations with IEC monolayers. As before, the total number of free trophozoites was expressed as a percentage of trophozoites at T_0_. Difference in rates of attachment was assessed for statistical significance using an unpaired t-test at each hourly time point, with a p-value ≤ 0.05 considered significant.

### Protein extraction and digestion

Trophozoites were detached from the HT-29 monolayer or flasks by briefly chilling the culture flask, collected by centrifugation and washed once with ice-cold PBS. Microscopy of detached trophozoites post host-cell interaction was performed to ensure absence of IEC contamination. Trophozoites were extracted in ice-cold SDS sample buffer containing 1 mM EDTA and 5% beta-mercaptoethanol, and then reduced at 75 °C for 10 min. Protein extracts were centrifuged at 0 °C at 13 000 × *g* for 10 min to remove debris, and stored at −20 °C.

Protein extracts were reduced with 5 mM dithiothreitol and then alkylated with 10 mM iodoacetamide. Alkylation was quenched with 5 mM dithiothreitol. Protein extracts were precipitated using methanol/chloroform^62^, followed by resuspension in 8 M Urea in 50 mM Tris (pH 8.8). The concentration of protein in each triplicate sample was measured by BCA assay (Pierce) before fractionation and digestion, initially with Lys-C overnight at 30 °C at a concentration of 1 μg enzyme to 100 μg protein. Lys-C digestion was followed with a sequential digestion with Trypsin at 37 °C with 1 μg enzyme for 100 μg protein. Digestion with trypsin was performed at 37 °C for 6 hours. Samples were acidified with trifluoroacetic acid, and then desalted on a 200 mg C18 SepPak (Waters, Massachusetts). Protein extracts were dried down using a vacuum centrifuge, resuspended and peptide concentration determined using micro BCA (Pierce).

### TMT labelling

Samples for TMT labelling were resuspended in 200 mM HEPES (pH 8) and a total of 70 mg from each triplicate for each of the 3 treatments, as well as a pooled control, were labelled in a 10plex TMT reaction (Thermo, San Jose, CA) using 0.14 mg of each reagent. Labelling was performed for 1 hour at room temperature and then quenched with 5% hydroxylamine. Each of the 10 samples were then combined, dried down using a vacuum centrifuge, reconstituted in 1% formic acid, and desalted on a 200 mg C18 SepPak (Waters, Massachusetts). The combined sample of TMT labelled peptides were dried down again and reconstituted in 1% formic acid prior to fractionation by strong cation exchange (SCX) high pressure liquid chromotography (HPLC) using a PolyLC PolySulfoethyl A (200 mm × 2.1 mm × 5 μm, 200 Å) column and UV detection at 210 nm. Samples were resuspended and initially loaded with buffer A (5 mM KH_2_PO_4_, pH 2.7, 25% ACN), and fractionated with a linear gradient of 10–45% buffer B (5 mM KH_2_PO_4_, pH 2.72, 350 mM KCl, 25% ACN) for 70 minutes, which was rapidly increased from 45–100% buffer B for 10 minutes at a flow rate of 300 μl/min. A total of 36 fractions of varying volumes were collected and dried down by vacuum centrifugation, before being combined to 12 fractions based on peptide content. These 12 fractions were desalted using C18 OMIX® tips (Agilent), dried down using a vacuum centrifuge and reconstituted in 1% formic acid in preparation for nanoflow liquid chromatography tandem mass spectrometry (NanoLC-MS/MS).

### Nanoflow LC-MS/MS for TMT labelling

Samples were analysed on a Q Exactive Orbitrap mass spectrometer (Thermo Scientific) coupled to an EASY-nLC1000 (Thermo Scientific). Reversed-phase chromatographic separation was carried out on a 75 μm i.d. × 100 mm, C18 HALO column, 2.7 μm bead size, 160 Å pore size. A linear gradient of 1–30% solvent B (99.9% ACN/0.1% FA) was run over 170 minutes. The mass spectrometer was operated in the data-dependent mode to automatically switch between Orbitrap MS and ion trap MS/MS acquisition. Survey full scan MS spectra (from *m*/*z* 350 to 1850) were acquired with a resolution of 70,000 at *m*/*z* 400 and an AGC (Automatic Gain Control) target value of 1 × 10^6^ ions. For identification of TMT labelled peptides, the ten most abundant ions were selected for higher energy collisional dissociation (HCD) fragmentation. HCD normalised collision energy was set to 35% and fragmentation ions were detected in the Orbitrap at a resolution of 70,000. Target ions that had been selected for MS/MS were dynamically excluded for 90 sec. For accurate mass measurement, the lock mass option was enabled using the polydimethylcyclosiloxane ion (*m*/*z* 445.12003) as an internal calibrant.

### Database searching for protein/peptide identification

For peptide identification, raw data files produced in Xcalibur software (Thermo Scientific) were processed in Proteome Discoverer V1.3 (Thermo Scientific) prior to Mascot searching against the Giardiadb.org 5.0 release for Assemblage A1, Strain WB (ATCC 50803). For searching, the MS tolerance was set to ±10 ppm and the MS/MS tolerance to 0.1 Da. One missed cleavage was allowed and carbamidomethylation of cysteines was set as a static modification. TMT 10plex modification of peptide N-termini and lysine residues, methionine oxidation, and deamidation of asparagine and glutamine were set as variable modifications. Search result filters were selected as follows; only peptides with a score >15 and below the Mascot significance threshold filter of *p* = 0.05 were included and single peptide identifications required a score equal to or above the Mascot identity threshold. Protein grouping was enabled such that when a set of peptides in one protein were equal to, or completely contained, within the set of peptides of another protein, the two proteins were contained together in a protein group. Quantitative information calculated from reporter ion intensities was only accepted for peptides with scores equal to or above the Mascot homology score, and the median value was taken to compare protein ratios.

### Analysis of differentially expressed proteins

The relative quantitation for host-cell interactions were derived by the ratio of TMT labels for each of the treatments over their respective replicate control (i.e. HSF R1 against Con R1 and CI IEC R1 against Con R1). A total of three expression ratios were derived for both HSF and CI IEC biological replicates, and an average fold change was calculated for each protein identified. In addition to TMT ratios for differential expression, proteins were analysed statistically via one-sample t-test to evaluate significance of observed protein expression changes. Proteins were only considered differentially expressed if they met both fold change criteria as well as ≤0.05 p-value significance. Functional annotation of proteins was performed using Uniprot to assign GO function, subcellular localisation, Interpro protein domains and structure annotations where available.

Prediction of secreted proteins was analysed using a series of bioinformatics tools to assess subcellular localisation^63^, presence of signal peptides^64^, transmembrane helices^65^ and nuclear localisation^66^. Proteins were submitted to TargetP v1.1 using the default settings for the algorithm for non-plant sequences, with a reliability score ≤3 selected for cutoff (with 1 being the highest reliability score). For analysis of signal peptides, proteins were submitted to SignalP v4.01, with the default settings for eukaryotic sequences. The presence of transmembrane helices was predicted using THMHH Server v2.0. Finally, as an exclusionary tool, proteins were submitted to NucPred to assess nuclear localisation signals with proteins above a prediction reliability score of ≥0.90 considered significant.

Several additional statistical analyses of the TMT dataset were performed to evaluate the variability of the dataset, and establish the presence of an underlying biological difference between HSF and CI treatments compared to control. Details of the additional statistical analyses can be found in Supplementary Methods. These include an assessment of sample variability based on control/control ratios, an unsupervised multivariate principal component analysis (PCA), and an analysis of the p-value distribution using paired t-tests between triplicates of HSF/Control and CI/Control ratios.

The mass spectrometry raw data files, database search results and TMT labelling protein quantitation results have all been deposited to the ProteomeXchange Consortium^67^ via the PRIDE partner repository with the dataset identifier PXD002398.

## Additional Information

**How to cite this article**: Emery, S. J. *et al.* Induction of virulence factors in *Giardia duodenalis* independent of host attachment. *Sci. Rep.* **6**, 20765; doi: 10.1038/srep20765 (2016).

## Supplementary Material

Supplementary Information

Supplementary Media 1

Supplementary Data S1

Supplementary Data S2

Supplementary Data S3

Supplementary Data S4

Supplementary Data S5

## Figures and Tables

**Table 1:**
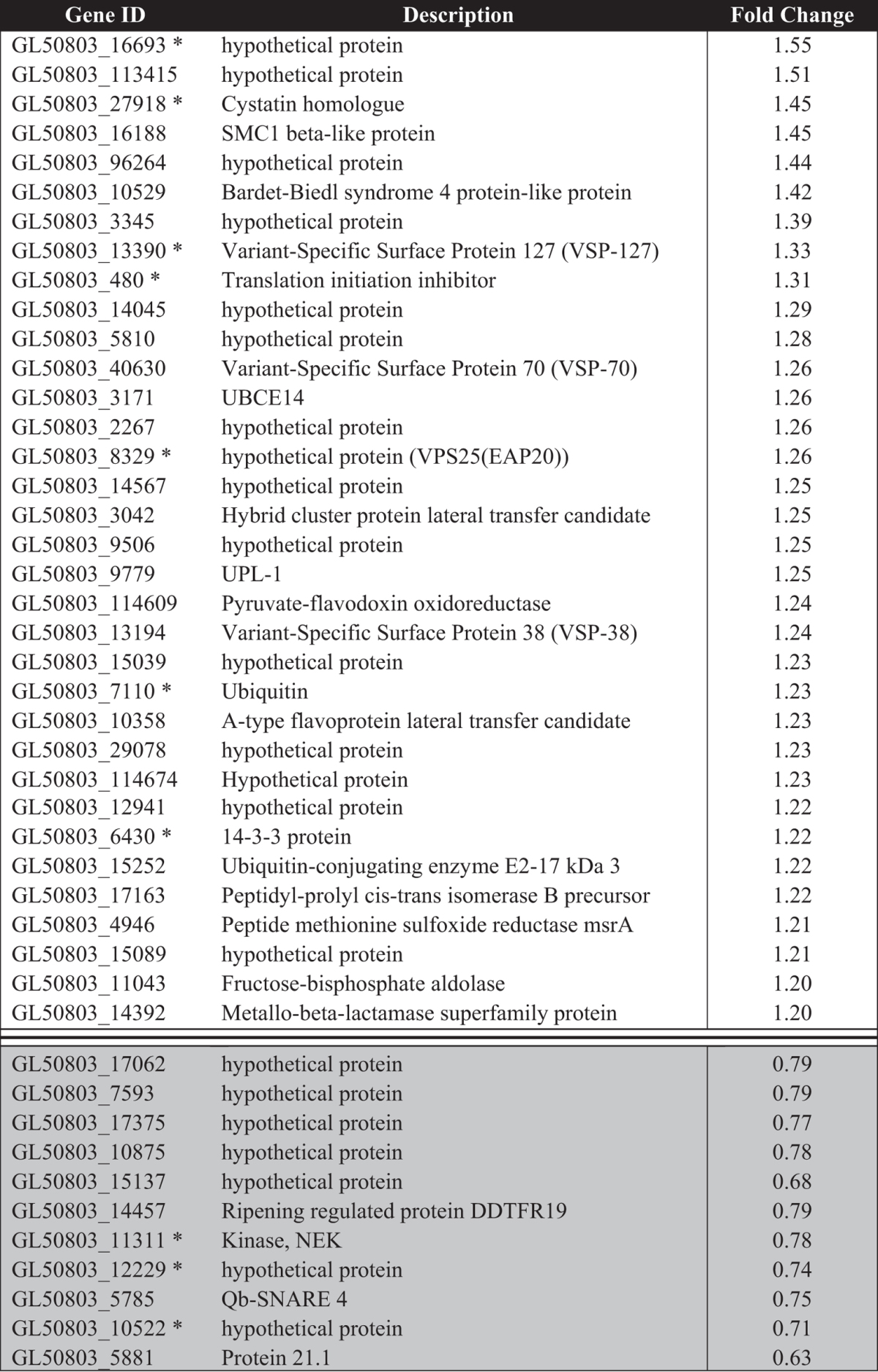
Differentially expressed proteins in *G. duodenalis* trophozoites co-incubated with HT-29 cells (IEC CI) for 6 hr in serum-free media.

Up-regulated proteins are designated by a ratio ≥1.2 also accompanied by a p-value ≤ 0.05. Down-regulated proteins are indicated with grey shading, and were designated based on a ratio of ≤0.8 which was accompanied by a p-value ≤ 0.05. Gene identifiers marked with a ‘*’ indicate a protein that was common between trophozoites co-incubated with the IEC monolayer and trophozoites incubated with HSF.

**Table 2:**
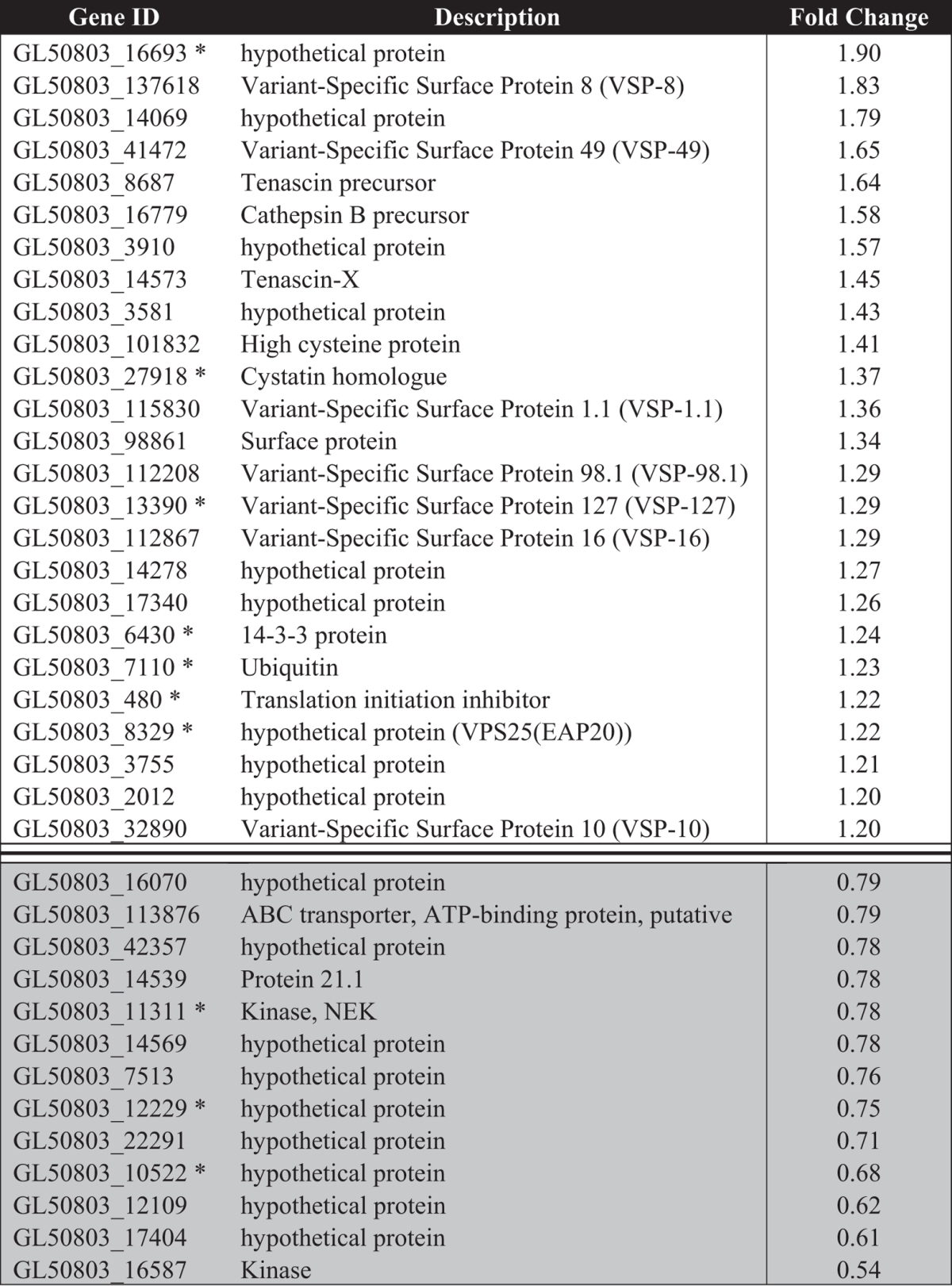
Differentially expressed proteins in *G. duodenalis* trophozoites incubated with host soluble factors generated from HT-29 cells for 6 hr in serum-free media.

Up-regulated proteins are designated by a ratio ≥1.2 also accompanied by a p-value ≤ 0.05. Down-regulated proteins are indicated with grey shading, and were designated based on a ratio of ≤0.8 which was accompanied by a p-value ≤ 0.05. Gene identifiers marked with a ‘*’ indicate a protein that was common between trophozoites co-incubated with the IEC monolayer and trophozoites incubated with HSF.
