# Supplementary material for: Induction of virulence factors in Giardia duodenalis independent of host attachment
Source: Sci Rep. 2016 Feb 12;6:20765. doi: 10.1038/srep20765 (PMC4751611; doi:10.1038/srep20765)
Supplement: Supplementary Information [file srep20765-s1.pdf]

<u>Supplementary Information</u>

Induction of virulence factors in *Giardia duodenalis* independent of host attachment

Samantha J. Emery[1], Mehdi Mirzaei[1], Daniel Vuong[2], Dana Pascovici[3], Joel M. Chick[4], Ernest Lacey[2], Paul A. Haynes[1*]

[1] Department of Chemistry and Biomolecular Sciences, Macquarie University, North Ryde, NSW 2109, Australia

[2] Microbial Screening Technologies, Pty, Ltd, Smithfield, NSW 2165, Australia

[3] Australian Proteome Analysis Facility (APAF), Macquarie University, North Ryde, NSW, 2109, Australia

[4] Department of Cell Biology, Harvard Medical School, Boston, Massachusetts, USA

* To whom correspondence should be addressed

Corresponding Author:

Professor Paul A. Haynes,

Department of Chemistry and Biomolecular Sciences, Macquarie University, North Ryde, NSW 2109, Australia

Email: paul.haynes@mq.edu.au

Phone: 61-2-9850 6258

Fax: 61-2-9850 6200

The following supplementary information is available as supplementary data files associated with this manuscript:

Supplementary Media 1: video showing trophozoites in control flasks (DMEM-only) compared to HSF-exposed trophozoites during the first 6-hour incubation in T75cm^2 flasks. Dark, tear-shaped trophozoites are adhered to the flask at low density, and continue to appear semi-motile against the flask wall during exposure to HSF. In contrast, control trophozoites in DMEM only are stationary on the flask wall at high density.

Supplementary Data S1: complete dataset for the attachment assay for trophozoites and HT-29 cells, including mean, standard deviations and p-values between co-incubation and control triplicates. Tab one contains the data for the adherence versus host-cell attachment assay, while the second tab contains the data for the HSF-exposure attachment assay.

Supplementary Data S2: Excel spreadsheet showing the complete protein identification and quantitation information for the TMT labelling experiment.

Supplementary Data S3: spreadsheet containing principal component scores (first tab) and loadings (second tab) resulting from a PCA analysis of the log2 (ratios) of all samples to the control pool, using all available quantitated proteins. The loadings were sorted in decreasing order of the first principal component, which shows a good separation of the control ratios from the rest; the top 5% highest loadings were highlighted.

Supplementary Data S4: Excel spreadsheet showing the complete functional annotation information for proteins up- and down-regulated during host-cell interaction analysis. Tables show the Giardiadb.org ORF number, protein description, fold change as well as GO annotation, subcellular localisation information and interpro protein domain/fold information. Up-regulated proteins in CI IEC and HSF are shown on tab 1 and 2, respectively, while the

down-regulated proteins in CI IEC interaction are on tab 3 and down-regulated proteins in HSF are on tab 4.

Supplementary Data S5: Excel spreadsheet showing the output from bioinformatics analysis of secreted proteins. Proteins were submitted to Target P for subcellular localisation, with a RI of ≤ 3 considered. Signal P was used to assess presence of a signal peptide, with a cutoff $\geq$ 0.5 considered for confident identifications and TMHMM used to detect presence of transmembrane helices. Finally, NucPred was used as an exclusionary tool for false positives in the secretory pathway, and scores ≥ 0.9 considered a hit for nuclear localisation. Output from analyses for up-regulated proteins in CI IEC and HSF are shown on tab 1 and 2, respectively, while the down-regulated proteins in CI IEC interaction are on tab 3 and down-regulated proteins in HSF are on tab 4.

The following supplementary information is included in subsequent pages of this document:

Supplementary Methods: statistical evaluation of TMT Dataset. A description of three additional statistical analyses of the TMT dataset and their results including, (1) evaluation of the data variability using control/control ratios (2) multi-variate principal component analysis, and (3) an assessment of the p-value distribution arising from paired t-tests between CI/control and HSF/control.

Supplementary Figure S1: histogram of standard deviations generated from triplicate ratios of $\log_2$(Control/PooledControl), based on all quantitated proteins in the dataset. Overlayed are the cumulative percentages of ratios with standard deviations lower than the respective bin. Approximately 95% of quantitated proteins have standard deviations less than 0.2.

Supplementary Figure S2: principal component scores plot in the space of the first three principal components generated for the whole dataset of $\log_2$ ratios of all samples with respect to the pooled control (label 131). The plot shows the control ratios well separated from the rest along the first principal component.

Supplementary Figure S3: histograms showing the distribution of p-values resulting from the paired t-tests comparing the Control samples respectively to the CI and HSF samples, underneath the volcano plots showing the p-values and fold changes. The p-value histograms have a peak corresponding to a larger number of low p-values, which is indicative of a real underlying effect; a random or noisy dataset is expected to generate a uniform distribution of p-values and hence a flat histogram.

Supplementary Figure S4: bioinformatic predictions of membrane and secreted protein analysis for up-regulated proteins. Distribution of secreted proteins in trophozoites incubated in HSF and trophozoites co-incubated with HT-29 IECs. Proteins were considered exported

to the membrane and/or secreted if they were positive for 1 of the 3 bioinformatics tools (TargetP, THMHH, SignalP) and negative for nuclear localisation (NucPred). The breakdown of the 11 proteins in HSF incubated trophozoites is further broken down in the column graph insert in the right. Complete summary of the predictive bioinformatics analysis can be viewed in Supplementary Data S4.

Supplementary Figure S5: design of host-soluble factor exposed non-specific adherence and specific host-cell attachment assay across 2 rounds of treatments. Trophozoites were raised in a single axenic culture and split between triplicates in three treatments. The first were trophozoites incubated in serum-free DMEM for 6 hours in the first round, and then co-incubated in the second round with confluent HT-29 (Con/CI). The second treatment exposed trophozoites to host soluble factors in serum-free DMEM for the 6 hours in the first round, and then co-incubated in the second round with confluent HT-29 also in the presence of host soluble factors (HSF/CI). The last treatment was a control for both rounds, and trophozoites were incubated in serum-free DMEM in both rounds of the assay (Con/Con). Non-specific adherence was measured in three timepoints (120 min, 240 min and 360 min) and specific host-cell attachment was measured hourly from 0-6 hours. Trophozoites were detached from the flasks used in the first round of the assay for non-specific adherence, and the same population was used to measure specific host-cell attachment.

Supplementary Table 1: Variant surface proteins (VSPs) identified across the TMT experiment, including their gene identifier, descriptor and fold change. TMT ratios above the 1.2 threshold have been bolded, while a shaded cell indicates a p-value ≥ 0.05. Only VSPs that were above the threshold for ratio and below the p-value for significance were considered differentially expressed. Interpro domains other than the 4 consistent VSP protein domains (Giardia variant-specific surface protein (IPR005127), Insulin-like growth factor binding protein, n-terminal (IPR009030), EGF-like (IPR000742) and Furin-like repeat

(IPR006212) domains) were considered for additional functional protein information, and listed where appropriate.

Supplementary Methods: Statistical evaluation of TMT dataset

We undertook an additional statistical analysis of the dataset from three separate angles: an evaluation of the data variability, a multi-variate look using principal components analysis, and an assessment of the p-value distribution arising from the paired tests undertaken. Taken together, these suggest that the experiment has low variability which justifies employing the differential expressions cut-offs selected, demonstrates good separation of the Control samples from the other two conditions, and illustrates a distribution of p-values that is consistent with an existing underlying effect.

1. Assessment of sample variability based on Control/Control ratios

The replicate variability of labelled experiments such as iTRAQ and TMT has been shown to be very low (such as in Karp *et al* [1]), and the same holds true in this experiment. Similar to the evaluation of Song *et al* [2] (specifically Figure 2 in Song *et al* [2]), we generated estimates of the variability of the control replicates using the log2-transformed ratios to the pooled control sample available from the TMT labelling experiment. Approximately 95% of the proteins have a standard deviation below 0.2, consequently in such cases a fold change of 1.2 ($\log_2(1.2) = 0.26$) would correspond to a z-score greater than 1 (Supplementary Figure S1). The standard deviations are, naturally, considerably lower than those determined in the evaluation of Song *et al* [2], since the experiments fits into a single TMT 10-plex labelling experiment.

2. Unsupervised multivariate Principal Component Analysis (PCA)

Using the log-transformed ratios of all samples to the control pooled sample (label channel 131), we visualised the Giardia samples using a principal component analysis undertaken on the whole dataset. The Control samples cluster together and are well separated from the Host Soluble Factors (HSF) and Co-incubation (CI) samples along the first principal component (Supplementary Figure S2). When considering the top 5% proteins with the highest loadings

for PC1, we find amongst them several of the differentially expressed proteins identified in the paired analysis in the manuscript (specifically, GL50803_3910, GL50803_27918, GL50803_13390, GL50803_10358, GL50803_6430, GL50803_14567, GL50803_9779, GL50803_17163, GL50803_42357). The principal component scores and loadings from this analysis can be found in Supplementary Data S3.

3. P-value distribution of paired tests undertaken

Pounds *et al* [3] shows that understanding the distribution of p-values is crucial to understanding whether the null hypothesis holds – in the case of a repeated test undertaken on an essentially random distribution, the p-value histogram is expected to be essentially flat, and the p-values are expected to be uniformly distributed; in contrast, where a real effect exists, the histogram will show a peak at the low end corresponding to lower p-values arising from real effects. In the case of this experiment, the p-value histograms obtained when comparing the two experimental states against the control are consistent with the existence of an underlying real effect (Supplementary Figure S3).

4. Additional References

1. Karp NA, Huber W, Sadowski PG, Charles PD, Hester SV, Lilley KS. Addressing accuracy and precision issues in iTRAQ quantitation. *Molecular & Cellular Proteomics* **9**, 1885-1897 (2010).

2. Song X, *et al.* iTRAQ experimental design for plasma biomarker discovery. *Journal of Proteome Research* **7**, 2952-2958 (2008).

3. Pounds SB. Estimation and control of multiple testing error rates for microarray studies. *Briefings in bioinformatics* **7**, 25-36 (2006).

Supplementary Figure S1:

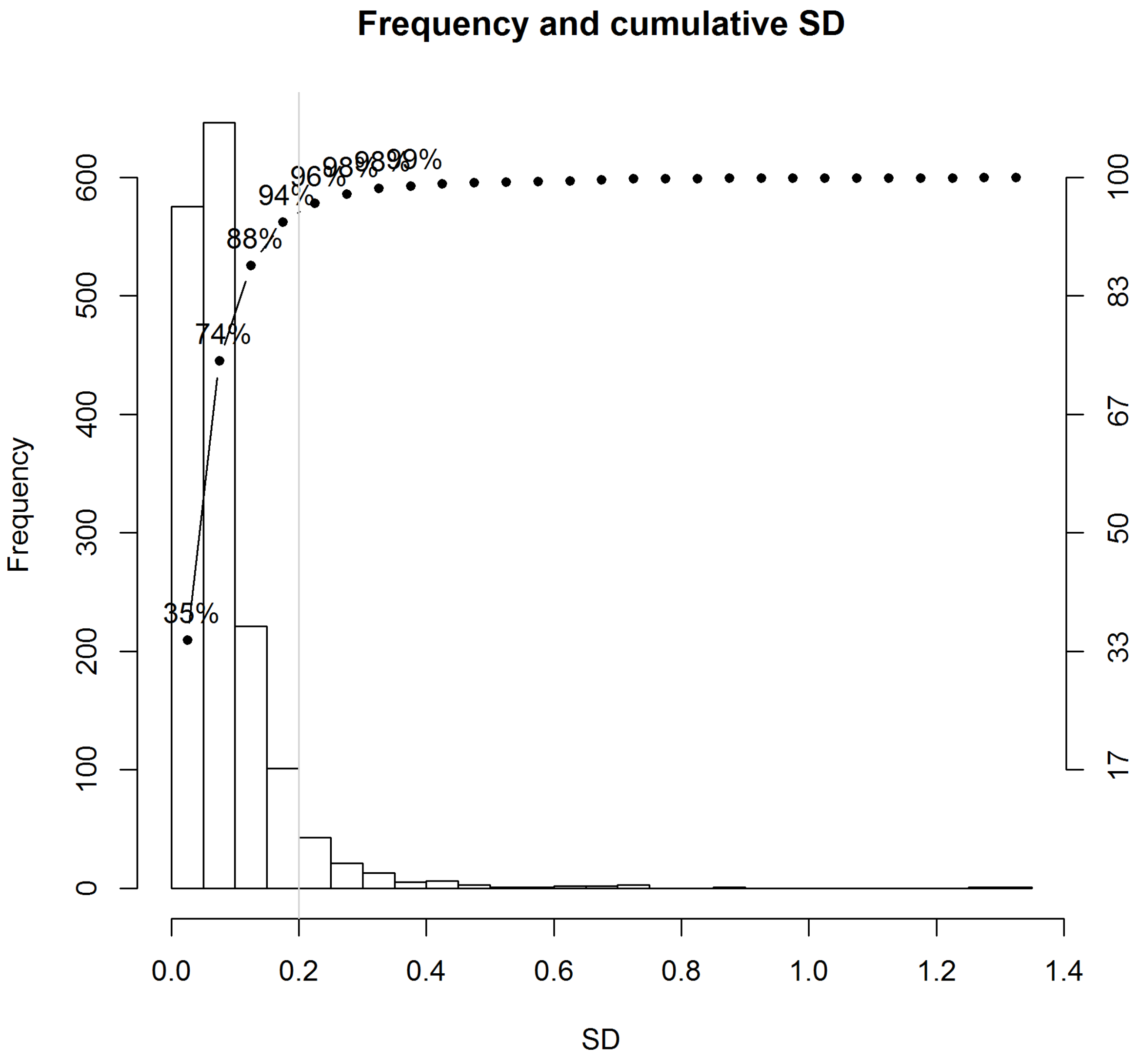

Supplementary Figure S2:

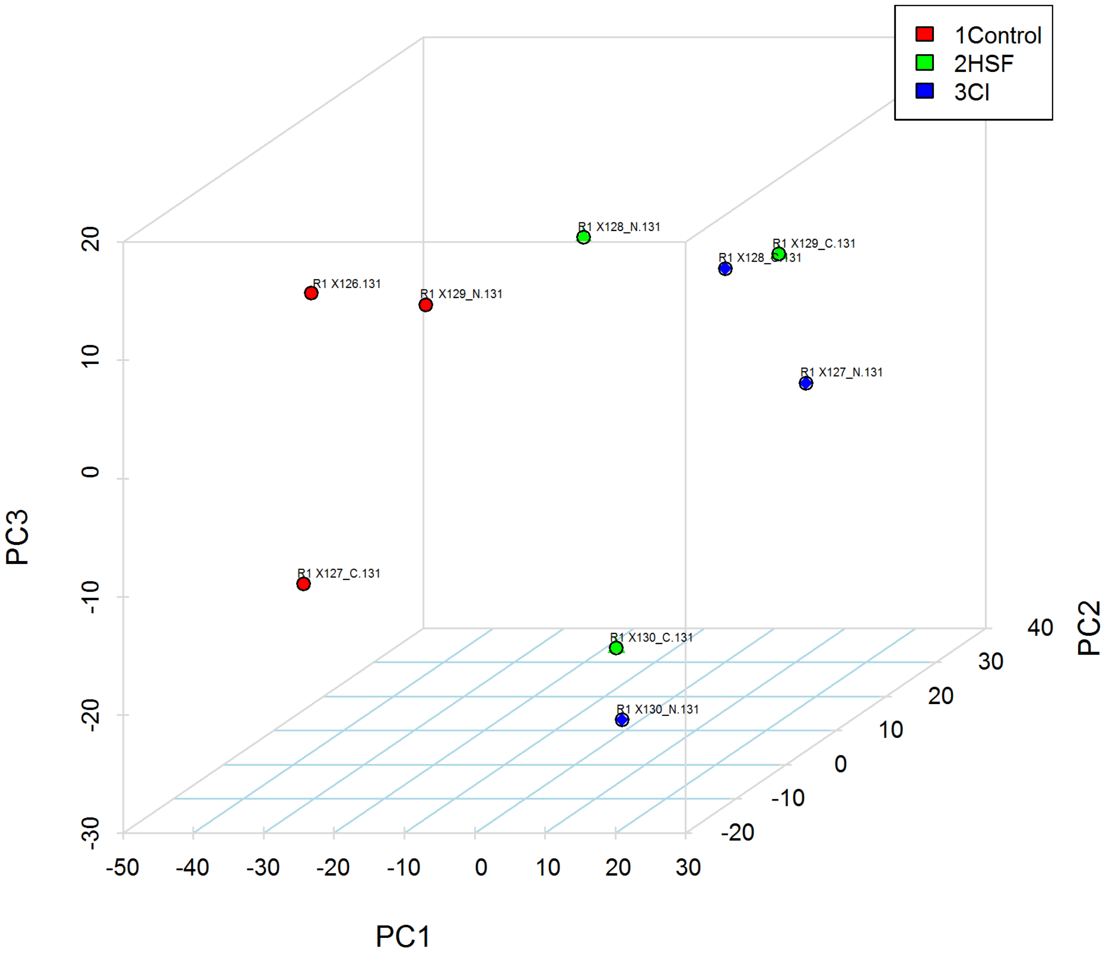

Supplementary Figure S3:

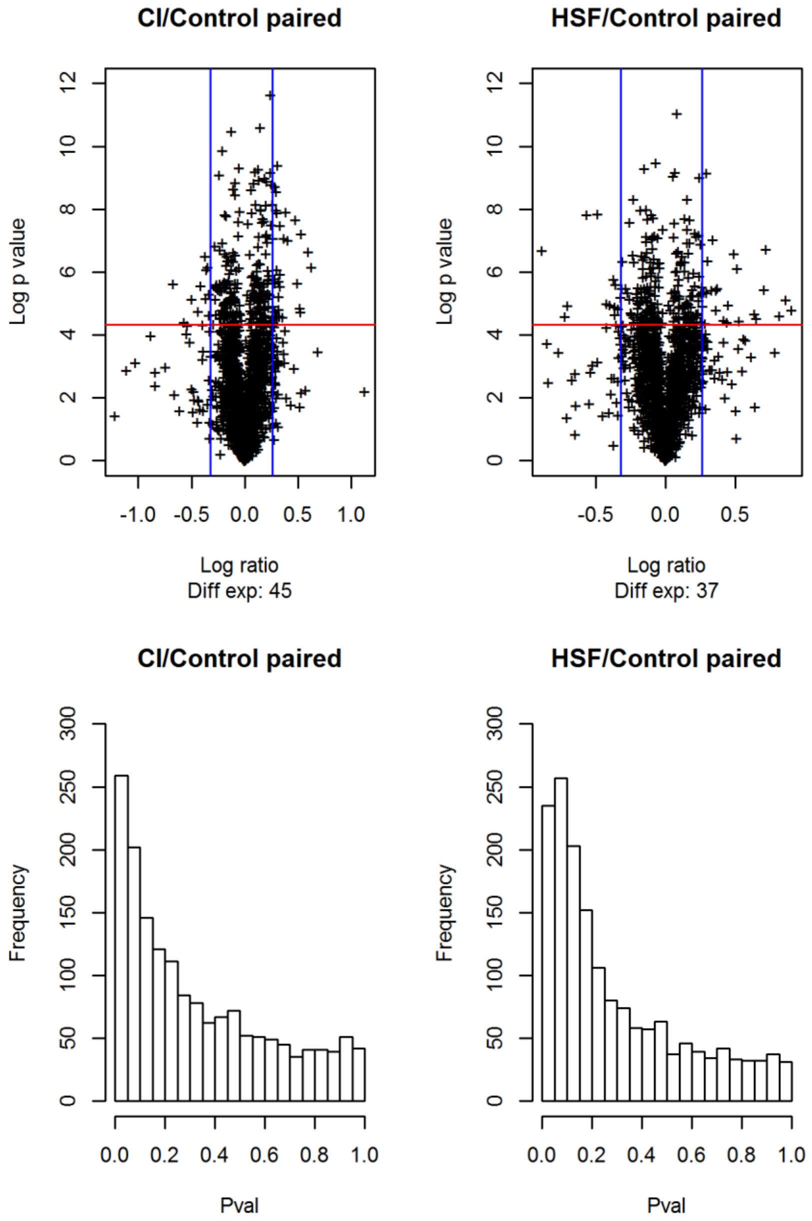

Supplementary Figure S4:

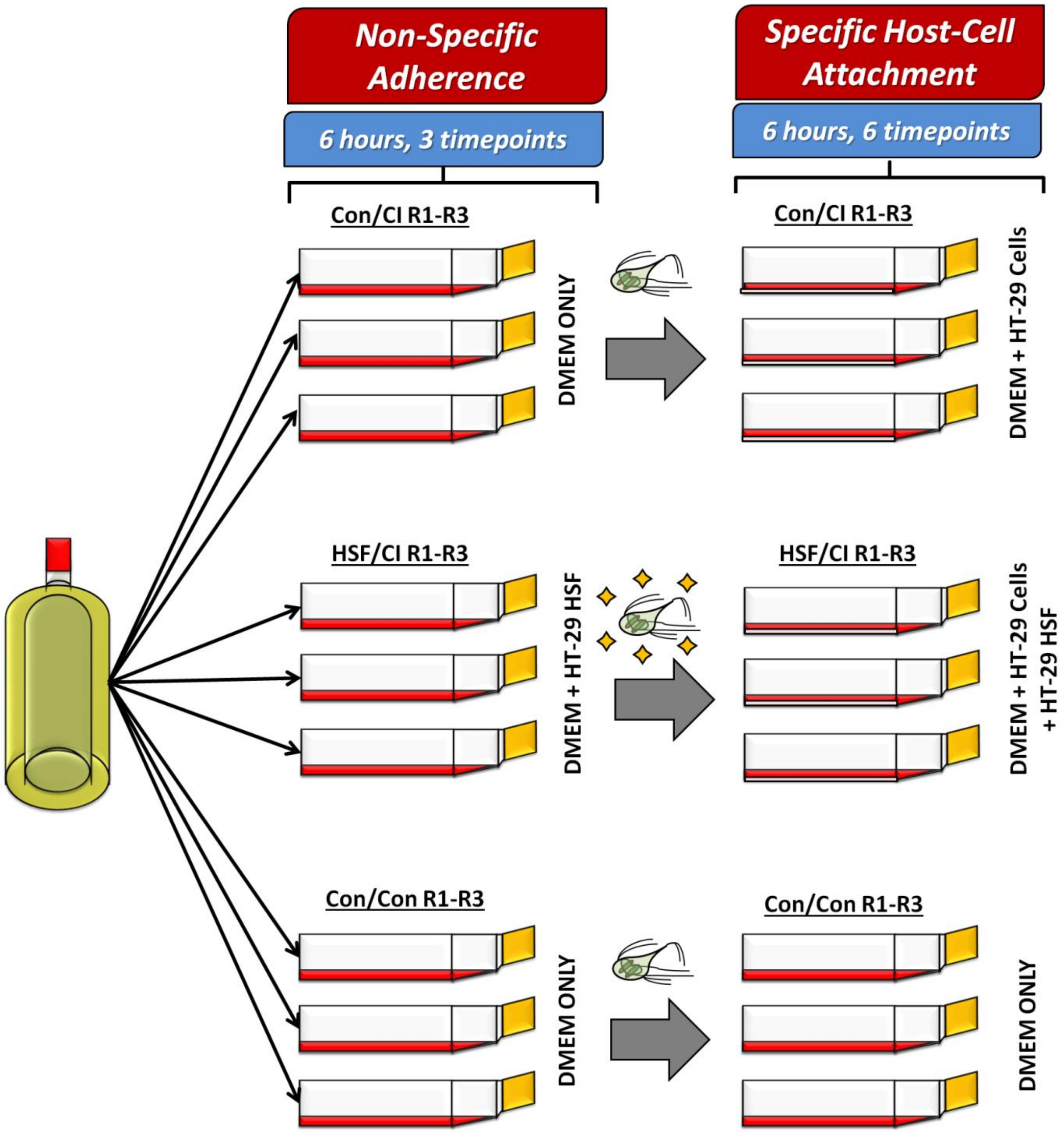

Supplementary Figure S5:

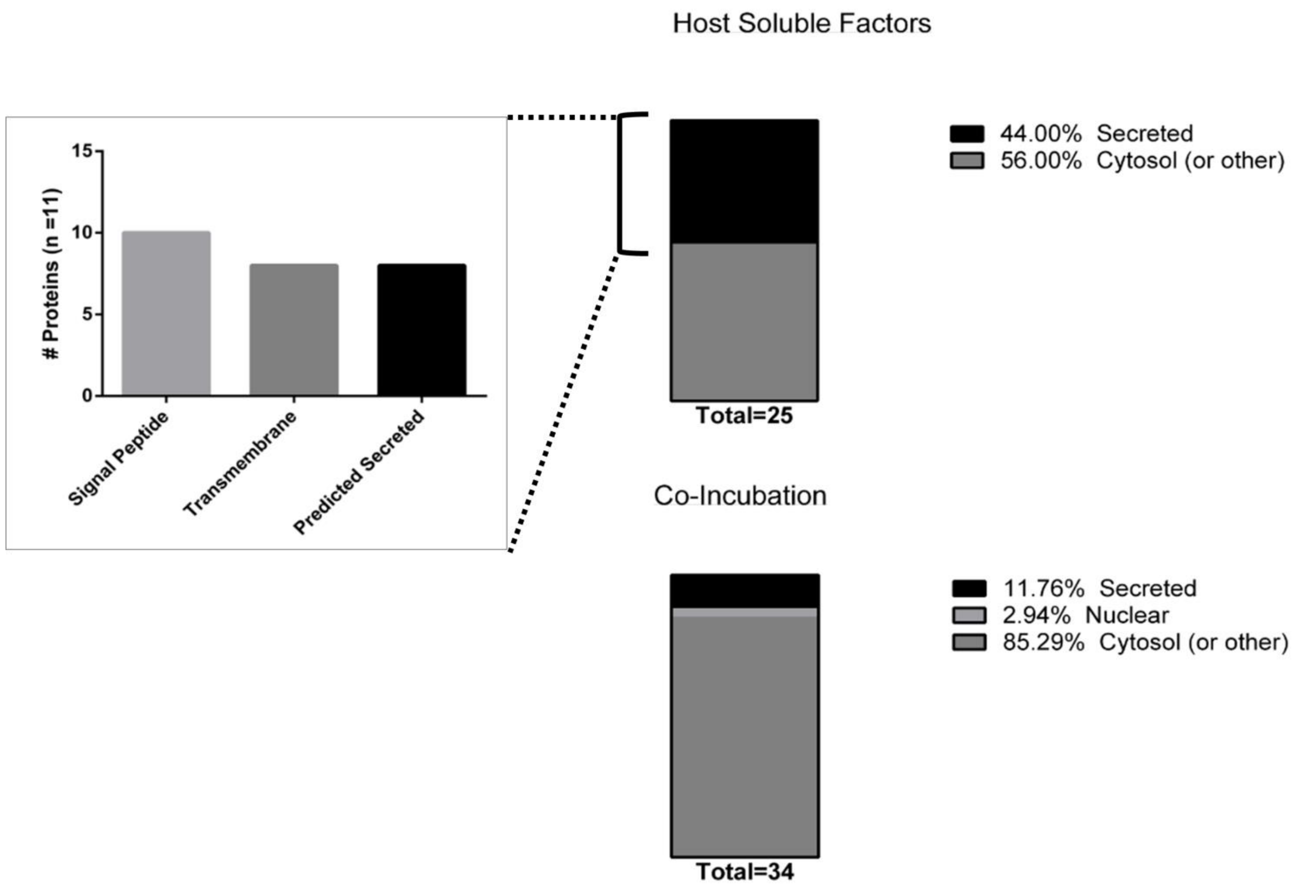

<u>**Supplementary Table 1:**</u>

Gene ID	Description	Fold Change		Addition Protein Domains
		6hr IEC CI	6hr HSF	
GL50803_137618	VSP 8	**1.65**	**1.83**	
GL50803_13194	VSP 38	**1.24**	**1.78**	
GL50803_41472	VSP 49	**1.53**	**1.65**	
GL50803_16472	VSP 52	0.91	**1.53**	
GL50803_136003	VSP 7.1	0.77	**1.51**	
GL50803_113163	VSP 29	1.18	**1.50**	
GL50803_115830	VSP 1.1	**1.21**	**1.36**	
GL50803_112208	VSP 98.1	**1.22**	**1.29**	
GL50803_13390	VSP 127	**1.33**	**1.29**	
GL50803_112867	VSP 16	1.02	**1.29**	BmKX domain (IPR015215)
GL50803_115742	VSP 31	**1.41**	**1.26**	
GL50803_34196	VSP 193	1.07	**1.21**	
GL50803_32890	VSP 10	1.31	**1.20**	Peptidase M8, leishmanolysin (IPR001577)
GL50803_40630	VSP 70	**1.26**	1.19	Peptidase M8, leishmanolysin (IPR001577)
GL50803_37093	VSP 25	1.19	1.16	
GL50803_13727	VSP 183	1.13	1.15	
GL50803_113439	VSP 45	1.13	1.13	
GL50803_101074	VSP 88	1.11	1.10	
GL50803_11521	VSP 126.1	1.16	1.10	
GL50803_113450	VSP 44	1.01	1.10	
GL50803_98861	Surface protein	1.13	**1.34**	Peptidase M8, leishmanolysin (IPR001577)
GL50803_119706	VSP 168.2	1.14	1.04	
GL50803_16158	VSP, putative	1.04	1.01	
GL50803_137723	VSP 26.1	**1.25**	1.01	
GL50803_137714	VSP 53.2	0.99	0.97	
GL50803_115797	VSP 54	0.99	0.95	
GL50803_101765	VSP 116	1.00	0.90	
GL50803_33279	VSP 100	0.92	0.85	
GL50803_113357	VSP 122	0.94	0.84	
GL50803_114674	Hypothetical protein	**1.23**	1.18	
